# Design considerations for the ideal low vision aid: insights from de-brief interviews following a real-world recording study

**DOI:** 10.1111/opo.12778

**Published:** 2021-02-02

**Authors:** Eugenie Golubova, Sandra D Starke, Michael D Crossland, James S Wolffsohn

**Affiliations:** 1 Aston Business School, Aston University, Birmingham, UK; 2 previously GiveVision, iCentrum, Birmingham, UK; 3 previously School of Engineering (Honorary Research Fellow), University of Birmingham, Birmingham, UK; 4 NIHR Biomedical Research Centre for Ophthalmology at Moorfields Eye Hospital and University College London Institute of Ophthalmology, London, UK; 5 Optometry and Vision Science, Aston University, Birmingham, UK

## Abstract

**Purpose:**

Low Vision Aids (LVAs) can have a transformative impact on people living with sight loss, yet the everyday requirements for developing such devices remain poorly understood and defined. This study systematically explored LVA requirements through a structured de-brief interview following a real-world self-recording study. The purpose of this work was to define the actual needs of those living with sight loss so that low vision services can better address them in future.

**Methods:**

Thirty-two visually impaired volunteers with varying levels of previous LVA experience participated in a de-brief interview centred around a structured questionnaire. The de-brief followed a one-week real-world study during which participants used recoding spectacles to capture and narrate all situations in which they would use a ‘perfect sight aid’. Content and thematic analyses were used to analyse interviews which had the purpose of contextualising these recordings and exploring requirements around psychological, functional and design factors.

**Results:**

Participants reported that 46% of tasks which they had recorded were most important to them. Of these tasks, 82% were encountered frequently. Few tasks emerged as very important across many participants, the remaining tasks reflecting individual lifestyles or circumstances. Every participant used at least one LVA in their everyday life and 72% identified further coping strategies. Current LVAs identified as consistently poor were distance LVAs, with all other devices receiving mixed or only positive feedback. Around two-thirds of participants would prefer LVA use on an ad-hoc / quick access basis rather than over long periods of time, and just over half would prefer to carry it rather than wearing it all day. Lack of consistency in these responses illustrated potentially different user clusters with divergent design needs. Two-thirds of participants emphasised the desire for a discreet LVA that does not attract attention. However, since half of all participants felt self-conscious in public or in front of other people when wearing the small recording spectacles, this may not be technically achievable.

**Conclusions:**

There is a substantial opportunity for new LVAs to address visual needs that traditional devices and coping strategies cannot support. Functional, psychological and design factors require careful consideration for future LVAs to be relevant and widely adopted.

## Introduction

Sight loss is set to become one of society’s major challenges in the near future owing to an ageing population and currently untreatable conditions such as dry age-related macular degeneration, myopic maculopathy and inherited retinal diseases. Hand magnifiers have been used by people with poor sight for centuries, and more sophisticated optical low vision aids (LVAs) have been prescribed by low vision clinics for around 50 years.^1,2^ Low vision aids can have a transformative impact on people living with sight loss.^3–6^ Advances in technology now enable the design of a new generation of wearable electronic sight aids.^7,8^ Positive signs of successfully utilising new technological capabilities also come from everyday consumer devices being increasingly utilised by and for people with visual impairment.^9,10^

In the past, studies on wearable low vision devices have focussed on assessing the impact of LVAs on visual performance dependent on the type of sight loss that they aim to alleviate (e.g., visual field expansion, obstacle avoidance or magnification),^11–16^ the tasks which require assistance^17–20^ as well as device use and health economic benefits.^21,22^ Initial design input requirements, especially in relation to functionality, ergonomics or usability, are typically of secondary interest. Research on LVA acceptance has focussed on usage indicators of current LVAs, but not on fundamental requirements. The disconnect between user needs and device design choices may be one reason for the limited uptake of LVAs, which has been noted for many years in both children and adults.^23,24^ In a recent review, factors leading to LVA abandonment included devices being too heavy, taking up too much space, suffering from poor ergonomics, being considered impractical for handling, limited technical performance, insufficient magnifying power, time-consuming operation, poor ease of use or substantial maintenance requirements.^24^ Particularly, LVAs for distance vision have been found to be abandoned or disused rather commonly.^25^ Without addressing user requirements, there is a risk that the next-generation of LVAs will also fail to meet the needs of those living with sight loss and will not be widely adopted.

To address this shortfall, there is a need for evidence-based design input requirements that guide future LVA development. Design of LVAs will benefit from such an evidence-based approach to align user needs and device specification. At the same time, following usability principles such as the universal design principles proposed by Story et al. more than 20 years ago^26^ will increase the likelihood of LVAs actually mapping onto the needs and capabilities of visually impaired end users from various demographics. Previously, we have reported outcomes of a real-world self-recording study in which visually impaired volunteers captured situations where they would use a ‘perfect sight aid’ by means of a spectacle-mounted video camera.^27^ The present article reports on a subsequent structured de-brief interview in which participants reflected on recordings and the experience of taking part in the study. The aim of this work was to capture the daily visual demands of people living with sight loss to understand desired activities in need of support, task characteristics and personal priorities. The objective was to support the user-centred design of future LVAs, whether head-mounted, hand-held, electronic or optical, to map more closely onto user requirements.

## Materials and methods

### Participants

This study adhered to the tenets of the Declaration of Helsinki and was granted a favourable ethical opinion and governance approval by the Aston University Ethics Committee (no. #1280). Participants provided written informed consent prior to commencing the study.

Thirty-two participants, recruited from the Aston Low Vision Clinic and GiveVision volunteer network, took part in the study. All participants had prior knowledge of and professional contact with one of these organisations. A deliberately broad definition of visual impairment for recruitment was used^28^ and 30 out of the 32 participants met the World Health Organisation's (WHO) definition of Low Vision^29^ on visual acuity grounds (visual acuity less than 6/18 and equal to or better than 3/60 in the better eye with best correction). Based on visual acuity, one participant met the WHO definition^30^ of mild visual impairment (6/12 to 6/18), eight had moderate visual impairment (6/18 to 6/60), 22 had severe visual impairment (6/60 to 3/60) and one met the criterion for blindness (< 3/60). Participants had varying levels of previous experience with optical and electronic LVAs. Recruitment was conducted over the phone using convenience sampling segmented into age groups (18–25 years, 26–64 years, 65+ years). One volunteer dropped out following the initial study sign-up without specifying the reason (they did not generate study data and thus were not included in the participant list). Refusals were not tracked.

As reported by Starke et al.,^27^ of the 32 participants, 15 were female and 17 were male; 26 participants still had vision in each eye and 6 had vision in one eye only; 16 participants had adult onset of visual impairment, 8 childhood onset and 8 had been visually impaired from birth. Mean (SD) age of participants was 47.5 (21.9) years (range: 18–87 years), binocular distance acuity was 1.2 (0.3) logMAR (range: 0.36 to hand movements (HM)), binocular contrast sensitivity was 0.8 (0.4) log units (range: 0.1–1.5 log units) and time since diagnosis was 22 (16) years (range: 2–64 years). Participant details are presented in *Table* 1.

**Table 1:** Participant details

N	Gender	Age	Sight loss condition	Onset	Years with sight loss	Visual acuity (both eyes open, logMAR)	Contrast sensitivity (log units)
1	M	30	Diabetic retinopathy	Adulthood	6–8	1.3	0.1
2	F	22	Leber’s hereditary optic neuropathy	Adulthood	4	1.68	0.30
3	F	27	Peter’s anomaly, glaucoma	From birth	27	HM	N/A
4	F	80	Dry macular degeneration	Adulthood	8	0.96	1.50
5	M	35	Aniridia	From birth	35	1.8	0.45
6	F	87	Age-related macular degeneration, cataract	Adulthood	12	1.4	0.60
7	F	25	Stargardt disease	Childhood	10	0.96	1.50
8	F	52	Dry macular degeneration	Adulthood	2	1.1	0.90
9	F	80	Dry macular degeneration	Adulthood	10	1.12	0.90
10	F	48	Uveitis, glaucoma, cataract, retinal detachment, aniridia	Childhood	40	1.16	0.8
11	M	21	Stargardt disease	Childhood	13	1.36	0.75
12	M	55	Retinal detachment	Adulthood	8	1.12	1.05
13	F	62	Sorsby’s fundus dystrophy	Adulthood	25	1.22	1.05
14	M	21	Blue cone monochromatism	From birth	21	1.12	0.65
15	M	19	Achromatopsia	From birth	19	1.3	0.90
16	M	19	Optic atrophy	Childhood	15	0.74	1.35
17	M	18	Microphthalmia	From birth	18	0.7	0.15
18	F	37	Diabetic retinopathy	Adulthood	10	1.36	0.75
19	F	20	Nystagmus, myopia	From birth	20	1.3	0.9
20	M	32	Retinitis pigmentosa	Childhood	20	1.06	0.15
21	M	50	Optic atrophy, nystagmus	Childhood	49	1.52	0.45
22	F	61	Glaucoma	Adulthood	16	0.36	1.15
23	F	54	Microphthalmia, nystagmus, congenital glaucoma	From birth	54	1.24	0.65
24	M	64	Albinism	From birth	64	0.96	1.35
25	F	66	Myopic macular degeneration	Adulthood	41	0.96	0.6
26	M	73	Central retinal vein occlusion	Adulthood	32	1.56	0.35
27	M	63	Dry age-related macular degeneration	Adulthood	8	1.34	0.60
28	F	75	Dry age-related macular degeneration	Adulthood	10	0.8	1.05
29	M	85	Dry age-related macular degeneration (left eye), wet age-related macular degeneration (right eye)	Adulthood	5	1.02	1
30	M	48	Retinoblastoma	Childhood	48	1.3	0.65
31	M	46	Diabetic retinopathy	Childhood	38	0.74	0.15
32	M	46	Stargardt disease	Adulthood	14	1.2	0.90

Visual acuity (VA) was measured as binocular distance VA (best corrected, ETDRS logMAR chart), contrast sensitivity (CS) was measured with both eyes open (Pelli-Robson chart).

### Initial task

**Figure 1:**
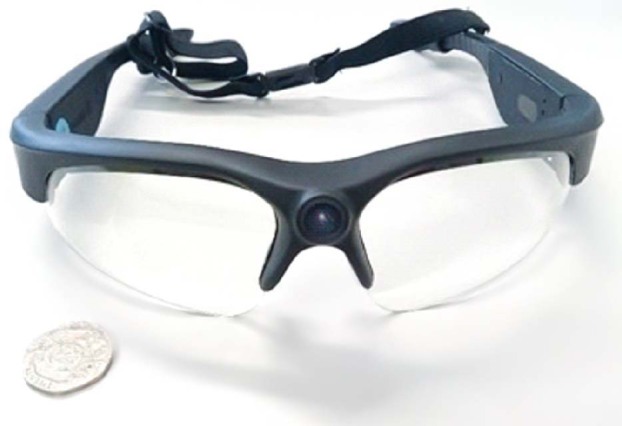
The SunnyCam Sport used in this study next to a 20 pence coin (£0.20) for scale (coin diameter: 2.14 cm).

The study task and methodological orientation have been described in detail previously.^27^ In summary, participants wore SunnyCam Sport video recording spectacles (https://lyte.uk) and were asked to record, for approximately one week, all situations during which they would use a ‘perfect sight aid’. The SunnyCam Sport (*Figure* 1) is a wearable camera with the design/shape of sports sunglasses. It was designed to be worn for recording physical activity. The device was chosen as it closely resembles the smallest design that could be achieved for a wearable LVA. This allowed us to explore feedback relating to psychological factors of wearing such a device without the cost of building a prototype of this size and shape - such development remains constrained by extensive costs and technological limitations.

Participants were instructed to capture the object of interest, narrate the intended activities and comment on the related difficulty. They were asked to record all scenarios with which they struggled, even if they had an LVA or coping strategy that would make the task accessible. This paper reports findings from a structured interview conducted as part of the de-brief at the end of the one-week recording study.

### De-brief questionnaire and interview

At the end of the study period, participants returned for a de-brief, which included a bespoke structured questionnaire. The questionnaire was developed around existing questionnaires in the literature, key study questions and the team’s experience working with people who live with sight loss. Together with the study task, it was piloted with two low vision volunteers (no major adjustments were made). The questionnaire did not incorporate any prompts or guides. The 10 questions appear in the results section together with the responses.

Interviews aimed to contextualise recordings using content analysis to examine what sight aids and coping strategies were and were not considered useful by participants while exploring preferences for an ‘ideal’ (electronic) sight aid. The de-brief interview was conducted one-to-one with one of five interviewers (GiveVision research team, Aston Low Vision Clinic teaching fellows) based on availability. All interviewers had many years of professional experience in interviewing and working with low vision patients and came from broad professional backgrounds. The debrief took place at the Aston Low Vision Clinic or GiveVision community spaces. Every interviewer was briefed not to ask leading questions or introduce bias through suggestions.

Participants were given the opportunity not to answer any questions with which they felt uncomfortable. No field notes, audio or video recordings were required during the interview. However, answers were captured in writing throughout the interview. Interviews were not time-limited, thus their duration varied. Participants were asked if they wished to review their responses, however none requested this option.

Transcripts were independently reviewed by the authors Golubova and Starke. No repeat interviews were carried out since every participant provided responses to every question (including ‘no further comment’ responses). Data saturation was discussed: no additional data was deemed necessary given the objective of interviews to contextualise existing recordings of the prior real-world study and the fact that every participant took part in the de-brief session. The study achieved a 100% response rate.

### Data analysis

Content analysis was used to analyse each participant’s response to each question by the author Golubova in Excel 2010 (www.microsoft.com) and SPSS 2013 (www.ibm.com). Themes/codes were derived from the data. If a participant diverged from the topic and started answering another question, these responses were re-mapped accordingly. In two instances where a response did not map onto the question or any other questions, the information was omitted from further analysis. Thematic analysis was used to group similar responses into categories for each question. These were counted, sorted and reported by frequency. The theme ‘other’ included responses that appeared only once per question and did not match any of the thematic categories. All identified themes and sub-themes were reported; thus, the coding tree was equivalent to reported results. All major and minor themes, as defined by the proportion of unique participants, were presented in the paper. Participant quotes identifiable by age and gender were presented to illustrate responses to each question.

## Results

### Question 1: What situations that you recorded were most important for you? Why?

"I’d like to be able to see what I’m looking at (…), [the staff] that work there [at the shop], they help but it’s not the same as being able to do it yourself. I went to a shop, I wanted a new washbag two weeks ago. Didn’t get one as I couldn’t find it. I [already] asked the lady twice for something else so I didn`t want to ask her again."

63-year-old male participant.

The majority of participants (N = 26, 81%) mentioned more than one situation which they recorded as being the “most important” for them. These situations were categorised into ‘specific activity’, ‘environment’, ‘lighting/clarity’ and ‘other’ (*Table* 2). Most commonly, the most important situations fell into the ‘specific activity’ category (N = 20, 63%; see *Table* 2 for a full breakdown). This was followed by the ‘environments’ category, requiring access, for example, to shops and unfamiliar places (N = 14, 44%). In the ‘specific activity’ category, reading any type of text was the most commonly named activity of importance, and 18 participants (56%) mentioned reading at least once. Overall, situations allocated to 'specific activity' (26 tasks in total) made up 46% of all mapped tasks in Starke et al. 27 based on the participants' scene recordings (56 tasks in total).

**Table 2:** Most important recorded situations (Question 1) and frequently encountered situations (Question 2) as named by participants

Count (percentage) of participants	Question 1			Question 2
	Specific activity (N = 20)	Environment (N = 14)	Lighting and clarity (N = 4) and Other (N = 2)	Frequently encountered situations
7 (22%)	Read package labels	-	-	-
5 (16%)	Read signs	Shop	-	Read (unspecified); Read package labels; Use public transportation
4 (13%)	Read print on TV; Reading (material unspecified); Use appliance dials, buttons and remotes	Unfamiliar places	-	Prepare meals; Watch TV
3 (9%)	Identify medicine	-	-	-
2 (6%)	Use ATM; Cross street; Find something on a crowded shelf; Prepare meals; Read books; Read menus; Use PC; Watch TV	Bus stop	Low light	Identify medicine; Read newspapers; Read timetables; Shopping; Use PC
1 (3%)	Appreciate environment; Avoid collisions; DIY; Get around outdoors in places you know; Groom yourself; Match clothes; Read newspapers; Read documents; Read notices; Read recipes; Read timetables; Recognise people from across the room; Search visually	Church; High street; Indoors; Kitchen; Outdoors; Out of home; School; Tube; Work	Glare; Lack of clarity; Parenting; all of the named activities	Appreciate environment; Cross street; Get around in low light; Glare; Groom yourself; Household chores; Judging distances; Read documents; Read maps; Read notices; Read signs; Search visually; University; Use ramps; Use a remote; Work

Full breakdown of responses across 32 participants. Multiple responses per participant were allowed. Answers for identical counts are separated by a semicolon.

PC, personal computer.

The reason for selecting situations as most important included: inability to do these activities/tasks at all or well enough (N = 24, 75%), not being possible or not always being possible to use a LVA or coping strategy (N = 10, 31%), personal safety/health reasons (N = 5, 16%), LVAs/coping strategy not convenient (N = 4, 13%), being dependent on the LVA (N = 2, 6%), a desire for greater independence/fewer limitations (N = 2, 6%) and a desire to have an LVA for a specific activity (N = 1, 3%).

### Question 2: What situations that you recorded do you encounter most frequently in everyday life? How often?

"I’d be more adventurous with cooking if I was more confident with what I’m seeing."

50-year-old male participant.

Of 58 named individual situations, participants described 82% as ‘frequent’ (e.g., daily, most of the time etc.). The frequency of situations/activities in daily life varied substantially between participants (*Table* 2), with unspecified reading, reading package labels and use of public transport named most commonly across participants (five participants each, 16%).

### Question 3: How did wearing the glasses make you feel? Why?

"Very comfortable. And I went around saying – this is great! This is great! Until my family said that they [the glasses] look weird."

48-year-old female participant.

Half of all participants (N = 16, 50%) mentioned that wearing the SunnyCam Sport made them feel self-conscious in public or in front of other people (*Table* 3). The most common reasons reported for feeling this way (*Table* 3) were conspicuousness of the camera and recording (N = 4), talking to one-self (N = 4) and drawing attention to oneself (N = 4). In contrast, 25% of participants (N = 8) reported that wearing the glasses was fine (“OK”, “normal”) or mostly fine except for certain situations or minor issues. *Table* 3 presents all general reported feelings and the individual reasoning for them.

**Table 3:** Feelings experienced as a result of wearing the glasses and reasons why as reported by the participants (Question 3)

Feeling (Count)	Reason why (Count)
Self-conscious in public (16)	Recording others/camera conspicuous (4) Talking to one-self (4) People ask questions / wonder what is going on (4) Standing out (3) Good for inside/environment where looks don’t matter (2) Family said glasses look weird (1) Bulkier than normal glasses (1) Embarrassed to meet someone they know (1)
Comfortable (9)	Lightweight/very light (4) Could forget they are on (2) Easy to use (2) Fit over own glasses (2) Comfortable though doesn’t like spectacles in general (1) Great field of view (1)
Fine / mostly fine (8)	Thick at the middle (1) Forgot that had them on once (1) Slight headache due to any wearable (1) Didn’t notice much (1) Similar to normal spectacles (1) Not uncomfortable (1) Used to wearable devices (1) No issues (1)
Uncomfortable (3)	Framing was difficult (1) Did not fit well (1) Over prescription specs (1)
Strange (2)	Talking to one-self (1) Doesn’t wear glasses normally (1)
Other (2)	Cool; protects eyes from the wind (1) Frame got in the way of remaining vision at the top (1)

Full breakdown of responses across 32 participants. Multiple responses per participant were allowed.

### Question 4: Would you wear glasses like this all day or rather carry them and use them when needed? Why?

"I’d use them when needed. This is what I do now with my other tools."

19-year-old male participant.

The majority of participants (N = 17, 53%) reported that they would rather carry the glasses with them and use them when needed (*Table* 4). These participants added that they would carry them in a bag, case or a pocket (N = 7) or around their neck (N = 2). Of the remaining participants, 16% (N = 5) would rather wear the glasses all day and 6% (N = 2) reported that either option was acceptable while 25% (N = 8) did not choose either option, instead reporting that usage would depend on circumstances and functionality of any potential LVA. Individual reasons for the four response categories are detailed in *Table* 4 where these were given.

**Table 4:** Reasons given why participants would wear the glasses all day or carry them with them as needed (Question 4)

Response to Question 4 (Count)	Reason why (Count)
Carry with me to use when needed (17)	Do not need them all the time (2) Discomfort (2) Heats up (1) Same as with other aids (1) Not used to wearing spectacles (1) Headache (1) Decreased field of view (1)
Depends on the circumstances (8)	All day if prescription accommodated (2) Home all day vs outdoors as needed (1) All day if high frequency of use (1) Can’t wear with the hat on (1) If comfortable for long use (1) Functionality that improves vision for more use cases (2) If instant – all day (1) If size small enough – all day (1)
Wear all day (5)	Convenient (3) Safer (less likely to break) (1) If prescription accommodated (1)
Either (2)	-

Use cases are defined as activities and actions that participants desired to undertake.

### Question 5: What is more important for you: quick access to a device for spontaneous tasks (minutes), or long tasks (hours)?

"Easily swapping among quick tasks with no need to reconfigure the device is important."

64-year-old male participant.

A total of 63% of participants (N = 20) considered quick access to a device for spontaneous tasks (minutes) more important, 28% of participants (N = 9) considered long tasks (hours) more important and 9% (N = 3) were unable to decide between those two options. As a way of explaining their preference, participants tended to focus on use cases, i.e., activities and actions that they desired to undertake. For example, some participants who preferred quick tasks mentioned use cases such as preparing meals, dressing, reading bus numbers, timetables, package labels and identifying medicine. Meanwhile, those with preference for long tasks highlighted university lectures, reading, watching TV and using a computer as use cases.

### Question 6: What would be the best thing about sight enhancement glasses [similar in design to the SunnyCam used in the study]?

"I would prefer to read [again], listening to everything is quite difficult."

27-year-old female participant.

**Figure 2:**
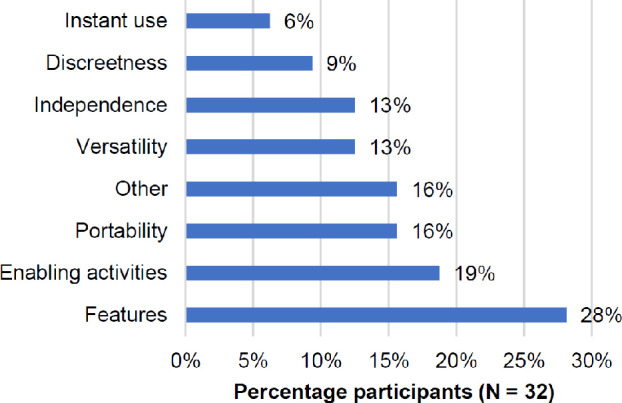
The best thing reported about sight enhancement glasses similar in design to the SunnyCam (Question 6) in order of reported frequency (multiple responses per participant were allowed).

The best thing reported about sight enhancement glasses with a design similar to the SunnyCam Sport used in the present study varied amongst participants (*Figure* 2), who also tended to mention more than one item. Most commonly (N = 9, 28%), participants reported various potential features: magnification (N = 4) as well as contrast/brightness adjustment, voice control, navigation cues, recording and artificial intelligence (each N = 1). The second most commonly reported item (N = 6, 19%) was enabling activities such as reading, recognising faces, activities requiring long distance vision, etc. Of the 16% of participants who provided unique “other” responses (*Figure* 2), these were: long battery life, comfort, outdoor use, replacement for a handheld magnifier and being able to wear the device more. Two participants responded with ‘don’t know’ to this question.

### Question 7: What would be the worst thing about sight enhancement glasses [similar in design to the SunnyCam used in the study]?

"If they don’t look like a normal pair of specs or sunglasses, they would label someone as sight impaired."

66-year-old female participant.

**Figure 3:**
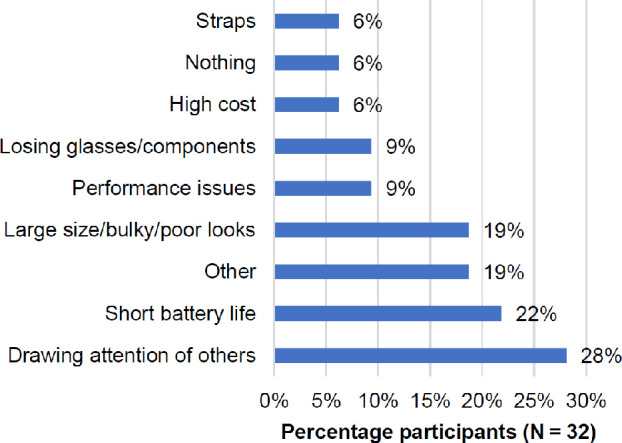
The worst thing reported about sight enhancement glasses similar in design to the SunnyCam (Question 7) in order of reported frequency (multiple responses per participant were allowed).

Most commonly (N = 9, 28%), participants felt that drawing the attention of others to their sight impairment would be the worst thing about an LVA with a design similar to the SunnyCam Sport (*Figure* 3). This was followed by 22% (N = 7) who mentioned a potentially short battery life and 19% (N = 6) who mentioned a potentially large size, bulkiness or poor looks. Other responses addressed potential performance issues such as low magnification, delay and overheating (N = 3, 9%), losing glasses or their components (N = 3, 9%), high cost (N = 2, 6%) and straps (N = 2, 6%). Two participants (6%) reported that “nothing” would be the worst thing assuming the device worked well. Three participants responded with "don’t know" to this question. Approximately 19% of participants (N = 6) gave unique responses categorised as ‘other’ (*Figure* 3): the device possibly not being portable, not accommodating prescription glasses, being easy to break, the frame getting in the way, experiencing anxiety about new technology as well as untruthful advertising.

### Question 8: What sight aids work for you and which don’t? Why?

"I have a telescope for looking at buses, but it draws attention to you."

48-year-old male participant.

**Figure 4:**
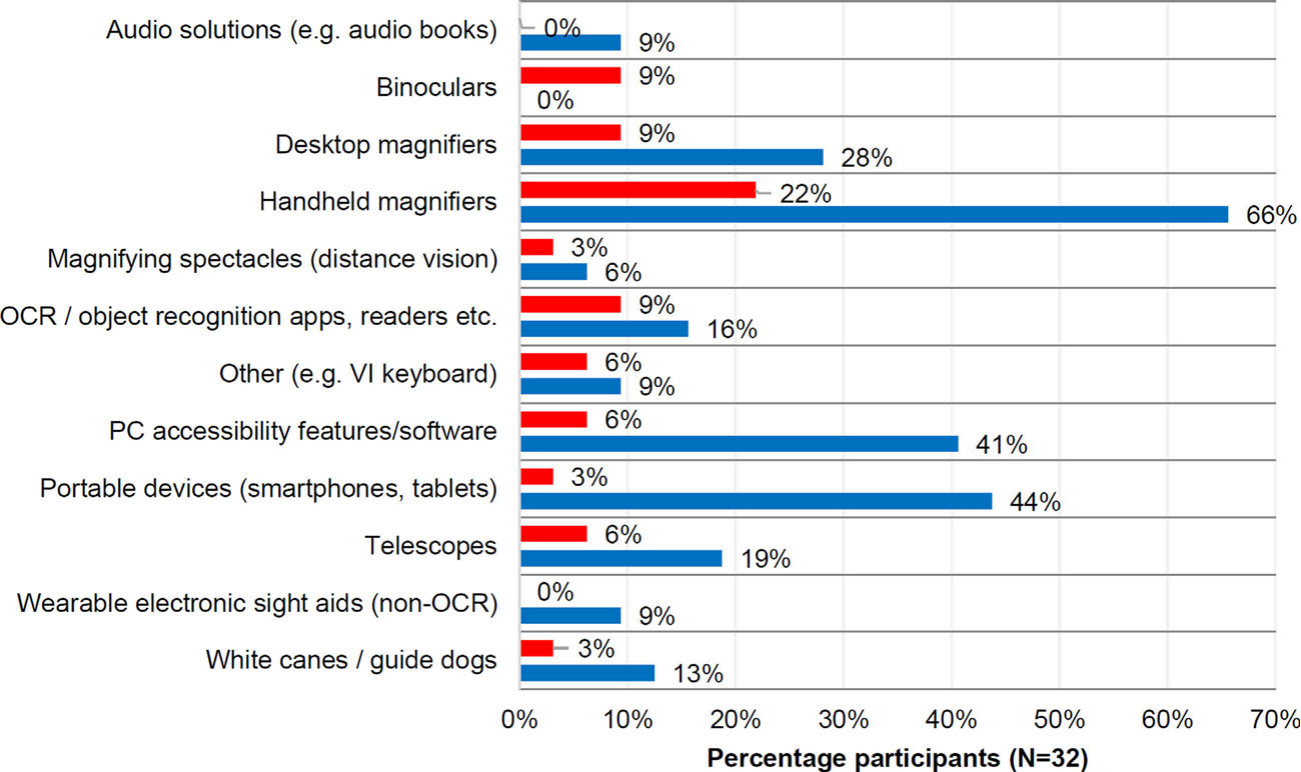
Sight aids which were reported to work (blue) or not work (red) well for participants (Question 8). OCR: optical character recognition. Devices are listed alphabetically

All 32 participants owned and used at least one LVA or other assistive solution, with a total of 87 specific items reported. On average, each participant owned more than two LVAs or assistive solutions. The most commonly reported sight aids (*Figure* 4) were handheld magnifiers, reported by 72% of participants (N = 23) with 75% of all comments being positive. The second most commonly mentioned sight aids were personal computer (PC) and/or laptop accessibility features or software (reported by 47% of participants (N = 15), with 87% of all comments being positive) and portable electronic devices such as smartphones/tablets (reported by 44% of participants (N = 14), with 93% of all comments being positive). Portable electronic devices usually referred to standard smartphones for a variety of uses such as taking photographs of objects of interest, zooming in with a camera and specific accessibility features. PC/laptop accessibility features ranged from screen magnifiers to voice controls for typing. Solutions used less frequently (*Figure* 4) included desktop magnifiers (reported by 38% of participants (N = 12), 75% positive comments), optical character recognition (OCR)/object recognition apps and readers. (reported by 25% of participants (N = 8), 63% positive comments), telescopes (reported by 25% of participants (N = 8), 75% positive comments) and assistive solutions such as white canes/guide dogs (reported by 16% of participants (N = 5), 80% positive comments). Binoculars were the only devices receiving exclusively negative feedback (N = 3, 9%) while audio solutions (e.g., audio books) and wearable sight aids received only positive feedback, albeit with small sample size (each N = 3, 9%).

The named advantages and disadvantages of the reported LVAs are summarised in *Table* 5 (excluding generic comments such as ‘it works’) and varied depending on the specific features of the LVA. However, some themes emerged on what participants regarded as desirable qualities. These were reliability of use and performance, especially for reading (N = 10, 31%), portability and on-the-go use (N = 8, 25%) and high or varying levels of magnification (N = 8, 25%).

**Table 5:** Reasons given why sight aids do and do not work for participants (Question 8)

LVA	Works because (Count)	Does not work because (Count)
Handheld magnifiers (optical, electronic)	On the go use/portable (7) High magnification, its levels (3) Easy to use (2) Easy to read (2) Task variety (1) Good for a quick read (1) Reliable (optical – battery doesn’t discharge) (1) In-built light (1) Inverts colour (electronic) (1)	Battery depleted (electronic) (2) Difficult to find place in the text (1) Weak magnification for small print (1) Flickers (electronic) (1) Loses focus (electronic) (1) No backlight (optical) (1) Too long to read (dome) (1)
Portable consumer devices (standard smartphones, VI smartphone, tablet)	On the go use (3) Varying magnification (3) Voice features (2) Inconspicuous (1) Large icons (1) Social media use (1)	Useless accessibility features (1)
PC/laptop accessibility features and software	Easy to use (1) Good magnification (1) Reliable (1) Enables PC use (1) Fast (1) Voice-to-text can ‘type’ anything (1)	Insufficient training to use (1) Losing place when reading with magnification (1) Slow reading on high zoom (1)
Desktop magnifier	Good for reading (2) Long use (1) Clear (1) Hands-free (1) High magnification (1) Touch screen (1)	Replaced by a wearable sight aid (1) Bulky (1) Dizzy when following magnified text (1)
OCR/object recognition apps, head-mounted aids and other devices	‘Reads’ everything/a lot or what’s needed (3) Accurate (1) Describes pictures (1) Nearly instant (1)	Unreliable accuracy (2) Not nice (1) No colour recognition (1)
Telescopes	Long distance (2) Adjustable focus (1) Good magnification (1) Maximum light (1) Works for different situations (1)	Conspicuous (2)
White cane		No need as has enough sight (1)
Guide dogs	Avoids obstacles (1)	-
Audio books	For book reading (1)	-
Head-mounted electronic low vision aids (non-OCR)	Enables an activity (2) Variety of use cases / places (1) Frequent use (1) Adjustable focus (1)	-
Binoculars	-	Heavy (1) If light, magnification too weak (1) Not clear (1) No use for small print (1)
Magnifying spectacles	Maximum detail (1) Good magnification (1)	-
Other (TV on-screen magnifier) (1)	Can sit further away from TV (1)	-
Other (voice recognition apps (1)	-	Poor voice recognition no matter how well articulated (1)
Other (magnifying shaving mirror) (1)	-	Weak magnification (1)

Numbers shown in brackets indicate the number of participants mentioning this reason.

### Question 9: What activities do you have a functioning coping strategy for and what is it?

"There’s a bus past me on the way to work and he [the driver] beeps to tell me he’s not the one [the bus I need]."

50-year-old male participant.

Activities with existing coping strategies (*Table* 6) were reported by 23 participants (72%), naming a total of 17 different activities. Most commonly, these included watching TV (19%), hobbies (e.g., bowling, archery, video gaming) and getting around in unfamiliar places (16% each). Various reading tasks were mentioned by 34% of all participants and commonly comprised bus timetables, bus numbers or package labels.

**Table 6:** Activities with functioning coping strategies (Question 9)

Count (percentage) of participants	Activity
6 (19%)	Watch TV
5 (16%)	Hobby (e.g., bowling, archery, gaming) Get around in unfamiliar places Read signs
4 (13%)	Read timetables “In general”
3 (9%)	Shop
2 (6%)	Read package labels Read street signs
1 (3%)	Find something on a crowded shelf Get around indoors in places you know Go to movies/theatre Read gym machine screen Read a whiteboard Read menus Use oven dials Use PC

Multiple responses per participant were allowed.

**Figure 5:**
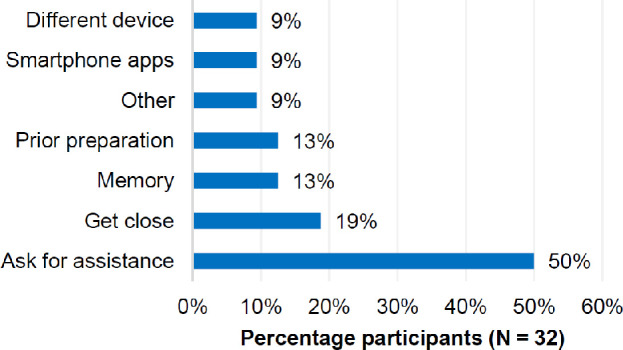
Reported coping strategies (Question 9) in order of reported frequency.

The most commonly reported coping strategy (*Figure* 5, N = 16, 50%) was asking for assistance, often from friends and family, but also from strangers. This strategy was most commonly reported for getting around in unfamiliar places, reading signs (e.g., bus numbers, directions) or package labels and ‘in general’. Getting close to objects of interest (named by 19% of all participants) was most commonly reported for watching TV (including video gaming) and cinema (sitting in the front row). Participants used various coping strategies for their hobbies that accounted for nearly all the ‘other’ responses and included high contrast (yellow) pins for bowling, laser pointers for archery or simply guessing.

### Question 10: What did you discover during the week that you were not aware of before?

"I subconsciously avoid doing things because I don’t want to attempt and struggle…. This aid [the ‘perfect’ LVA] would open my opportunities."

37-year-old female participant.

Seventeen participants (53%) reported having discovered something new that they had not been aware of before participating in this study. For 11 participants (34%), this centred around positive possibilities of a ‘perfect’ LVA: of these, seven participants (22%) noted use case(s) which they thought could be helped with a device (e.g., read food labels when cooking, gardening, TV, PC, looking at a clock, etc.) while four (13%) mentioned expected benefits (e.g., a multipurpose LVA instead of having to change between different aids when tasks change, hands-free control, etc.). The remaining six participants gave responses with a negative connotation: two participants (6%) realised they were self-conscious in public wearing even a small device like the SunnyCam, two participants (6%) felt that the study drew attention to their poor sight and difficulty with some tasks and two participants (6%) giving ‘other’ responses referred to thinking that a perfect LVA would not be of use to them and even wondering whether having full sight would have downsides.

## Discussion

This study reports findings from a structured de-brief interview contextualising a week of activity captured through a spectacle mounted camera by a cohort of visually impaired volunteers,^27^ taking their sight aids, assistive solutions and coping strategies into account. In this first study of its kind, participants had freedom to record and report any tasks they deemed important during the project, unlike other research which usually only focussed on one or several challenging activities (e.g., mobility, housework).^31–33^

### Activities of importance for people living with sight loss

In line with other studies on the impact of low vision,^34–37^ participants reported marked difficulties in their ability to engage in activities of daily living. Five tasks emerged as very important for many participants: i.e., reading package labels, reading signs, using appliance dials/buttons/remotes, finding something on a crowded shelf and using a computer. At the same time, many participants reported other activities that were just as important to them, and these should also be considered in LVA design to make devices more useful. It is important to note that computer use does not currently feature in many low vision activity questionnaires and needs to be incorporated. Similarly, Latham et al. found that retinitis pigmentosa patients named indoor and outdoor mobility, shopping and physical activity/sports as most difficult.^38^ In a 2007 study, Massof and colleagues divided visual ability into the two factors of reading and mobility function.^19^ Reading was a priority area in the present study, while mobility featured less prominently across activities, being centred around shopping trips, leisure or general travel. We identified areas related to everyday activities surrounding household, computer use and shopping as highly relevant, which should be considered in future LVA design.

Comparing the most commonly recorded tasks from previous self-recordings^27^ with the important situations recalled in the de-brief demonstrated strong congruence. In the de-brief, the most frequently named important tasks (as named by at least four participants) were reading package labels; reading signs; shopping; reading in general; reading print on TV; appliance dials, buttons and remote controls as well as dealing with unfamiliar places. Similarly, the top seven tasks by number of participants in Starke et al.^27^ were finding something on a crowded shelf; reading package labels; using appliance dials, buttons and remote controls; reading signs; watching TV; reading mail and cards and using the PC.

### Use of existing LVAs and other coping strategies

We found that traditional LVAs can provide substantial benefit under specific circumstances to many participants, but could not meet all key needs. These findings are consistent with other research indicating that the availability of current LVAs, assistive solutions, possible coping strategies and support from family/friends are not sufficient to enable participation in many activities of daily living for those with sight loss.^31^ We found that the same LVA may provide varying levels of benefit to different people, likely dependent upon the nature of sight loss and the desired tasks. The most commonly used sight aids were handheld magnifiers, portable electronic devices such as smartphones/tablets and computer/laptop accessibility features or software, all receiving mixed positive and negative comments. Less commonly used solutions also received mixed feedback. The only LVAs without mixed responses were audio solutions and wearable electronic LVAs which received only positive feedback (each N = 3) while binoculars received only negative feedback (N = 3). This could make a case for wearable LVAs addressing an unmet need for distance vision, as previously described in the literature.^25,39^ Since 32% of the activities recorded in this study required viewing targets positioned more than 1 meter away from the observer 27, LVAs facilitating distance vision may be taken up to a greater extent if they were built to meet these needs.

Despite every participant indicating that they owned at least one LVA or assistive solution, and the majority (72%) using additional coping strategies, participants recounted that they were unable to perform many activities that were most important to them satisfactorily. Indeed, 75% of participants stated that they considered tasks important because of their inability to perform them adequately, or even at all. Similarly, participants reported no available sight aid or coping strategy for 57% of the tasks captured through self-recording.^27^ When comparing questionnaire responses and captured scenes,^27^ only 66% of participants showed or mentioned an LVA at least once in the recordings, despite activities being considered as potentially suitable for existing LVAs. This is particularly unexpected because in 68% of recorded cases the object of interest was within reach^27^ and traditional LVAs are typically near vision devices. While participants might have chosen not to show the LVA in the recording, underuse of suitable LVAs may also be an issue.

### Should an LVA be designed for quick ad hoc access or being worn all-day?

In this de-brief, 63% of participants indicated that quick access to a device for spontaneous tasks (that take minutes) was more important to them than a device supporting longer tasks, comparing well with 75% of recorded scenes falling into the ‘ad hoc’ category of up to 5 min task duration.^27^ 53% of participants also reported that they would rather carry a device and use it as needed. This illustrates that ad hoc access to a device and associated features such as quick start-up and response times are important for LVA design. Importantly, activities such as cooking may require a succession of ad hoc tasks that require being hands-free, including reading a recipe, finding ingredients, assembling a kitchen tool and setting oven dials. The user may wish to wear the sight aid continuously for such tasks or use it only when needed, and this should be facilitated through the design of the device. For those participants preferring ad hoc access, reasons given were typically related to some negative aspect of wearing a LVA all day, such as discomfort, decreased field of view or an electronic aid heating up, as well as simply not needing the sight aid for every activity. Those participants for whom preferences depended on the situation typically reported that they would wear a potential LVA all day long if it had certain features, such as accommodation for their spectacle prescription, specific functionality, both comfort and response speed, or they would only use it under specific circumstances (such as at home). In other situations they would use it as needed. The key reason for participants preferring to wear the LVA all day was convenience.

### What features might result in LVAs adoption and what should be design priorities?

Three emerging themes for a ‘perfect’ LVA were: portability and on-the-go use, benefit of high/varying magnification and reliability (working when needed and performing well for desired activities). The most common and important tasks varied with regards to where they occurred, what challenges they required and the exact requirements for LVA design to facilitate them. An LVA aimed at facilitating most activities of daily living would need to be suitable for both indoor and outdoor conditions, adapt to different types of lighting (including artificial light) and be acceptable in public. An ideal electronic LVA would need good functionality for reading text in many circumstances at different light levels. This would require image stabilisation and fast zoom action to change between seeing ‘the big picture’ and details; there may also be scope for additional features such as text-to-speech functionality. The perceived importance of desired activities, rather than specifics of sight loss, may be a key determinant of participation of the elderly visually-impaired in various daily life activities.^40^ Future LVAs should facilitate engagement in these high-ranked activities.

Previous studies have proposed various design concepts following consultation with visually impaired users, sometimes arriving at opposing suggestions. For example, a spectacle-like device controlled through a wristband or ring emerged from a survey of 10 people.^41^ In contrast, the proposal of smart glasses was questioned in another study.^42^ In terms of processing power, a small delay (43.5 ms) in real-time processing was not perceived by participants unless they paid attention to it,^43^ yet it is unknown whether this is acceptable during walking or head movements. Battery life of under an hour has been cited as a significant device limitation in this and other studies.^44,45^ These desired features are in contrast with each other, as for example longer battery life may require a larger battery and hence bulkier size, unless the battery can be carried separately. This in turn would require wires/straps, which is not considered desirable. A 2019 review of factors related to the uptake of low vision aids^24^ found that devices perceived as too heavy, taking up too much space, suffering from poor ergonomics, impractical handling, technical performance, insufficient magnifying power, time-consuming operation, poor ease of use or substantial maintenance requirements were more likely to be disused and abandoned. This calls for human factors/ergonomics, usability and established medical device design standards becoming a priority in the design of LVAs. A clear challenge for the prioritisation of LVA design features will be the trade-off between functionality and appearance.

This work and others^9,10^ have illustrated the increasing uptake of consumer electronics as assistive devices for the visually impaired. However, consumer electronics and modern LVAs will likely utilise technology which may feel alien to older generations. The UK Royal National Institute for the Blind (RNIB) MyVoice study^31^ highlighted that while 86% of 18 to 29 year-olds “felt able to make the most of new technology”, 66% of those aged 75 and over did not even use a computer, the internet or portable devices such as smartphones and tablets. Designing for this demographic to overcome hurdles of technology adoption should be a priority, as would following the principles of inclusive design.

### What is more important, improvement in sight or device appearance?

Culham et al.^46^ found that a device’s impact on visual performance was the key predictor for higher subjective evaluation, rendering perceived comfort almost insignificant. On the other hand, the present study showed that a significant number of people may feel uncomfortable wearing a device even with the small size of the SunnyCam Sport sunglasses in public and may choose not to do so: the majority of participants (69%, N = 22) emphasised issues of self-consciousness and desire for discretion in front of others, especially strangers, when using wearable sight aids. Partly this could be attributed to the study objective of recording and narrating scenes, which drew attention if done in public. However, this also indicates that device features such as voice control, audio feedback, image taking as well as cosmetic appearance need to be carefully considered if an LVA is to be used in public. Similar to the desire for a discreet design found here, two small studies (N < 7) indicated a preference for a smaller wearable device or a see-through display for ergonomic and cosmetic reasons.^47,48^ However, this may not result in superior sight enhancement since see-through devices suffer from scene clutter, low contrast and light interference, potentially rendering such design aesthetically pleasing, but functionally inadequate. Looking ahead, employing pretotypes (the term for the approach we used in this study) and prototypes, as well as careful iterative user testing throughout the LVA design process would allow further exploration of these challenges. The stigma of using assistive devices in many other areas has been widely reported including hearing loss^49^ and wheelchair use^50^ as well as visual impairment.^51^ Minimising the potential for perceived stigma and self-consciousness should therefore be a guiding principle for design.

Currently, there is no LVA technology available that would offer the small design footprint of the SunnyCam Sport used in this study, with promises made by industry not living up to expectation over the last decade. At the same time, since even this minimalistic design was considered conspicuous by some participants, one key question is whether an ‘unnoticeable’ design is achievable technically, or whether psychological barriers to device adoption need to be addressed differently. In this context, a positive example is set by OpenBionics (www.openbionics.com), who re-defined limb prosthetics as a desirable fashion statement reflecting the owner’s personality rather than trying to make the prosthetic unnoticeable.

### Study limitations

During the debrief, three participants mentioned that they did not record all of the situations they wished to record, in particular when these were spontaneous and unexpected. Therefore, the study might have been biased towards more familiar situations. Reported coping strategies may not have encompassed those which participants use automatically without thinking, for example increasing lighting or getting closer to objects of interest. Future in-depth exploration of such coping strategies could prove valuable.

The present study included a measure of prioritisation and importance similar to the approach taken by Massof and colleagues.^17,18^ They developed a composite metric combining importance and difficulty. This could serve as a framework for future studies by prioritising or ranking functional LVA requirements. The present study did not request a specific difficulty rating from participants, which would make a worthwhile addition for future study designs.

### Summary and conclusions

Key findings of this de-brief addressed psychological, functional and design aspects of LVAs.

First, a prominent psychological consideration centred around LVAs making their users feel self-conscious. This was reported by half of the participants, especially if LVAs are perceived as conspicuous and/or label users as sight impaired. This theme was mirrored in the participants’ reported ‘worst thing’ about an LVA similar in design to the SunnyCam Sport, with 28% naming “drawing attention to oneself”. It is however important to recognise that while some people may feel strongly about this, others did not mind. It could therefore be worthwhile considering two different design solutions for individuals with sight loss, one making a compromise between performance and looks in order to be accepted, and the other focussing purely on performance for those users who do not mind the appearance of the device. Further user segmentation based on key needs might prove beneficial.

Second, evaluation of functional requirements illustrated that there may be a dissociation between task importance and frequency, where neither metric can be used in isolation to define design input requirements with regard to priority functions. Tasks involving reading were most often named with regard to importance and frequency, while tasks that reflected individual lifestyle choices received lower counts. This highlights that there are relatively universal requirements that LVAs should address (reading) while also accommodating a vast range of important personal interests and activities in order to be useful and relevant for a large number of people.

Third, in terms of design, the majority of participants considered quick device access to be more important than the ability to wear an LVA all day, mirrored by the preference to carry an LVA and use as needed rather than always wearing it. Again, it is important to note that a smaller fraction of participants were of the opposite opinion, while a significant number indicated situation specific preferences.

The logical next step in researching LVA design requirements would be cluster analysis and a segmentation exercise to specify LVAs for clearly defined user groups. This would help ensure that future devices live up to the demands of those using them. At the same time, based on these findings and those of others, low vision practitioners can be encouraged to think about personalised rehabilitation, similar to medical colleagues considering personalised medicines. Lack of knowledge concerning LVAs for those living with a sight condition is another challenge to the widespread adoption of LVAs, where improved outreach and awareness could positively impact their uptake. Finally, we encourage designers of LVAs to assess new products using qualitative and quantitative research methods, perhaps using an instrument such as the Quebec User Evaluation of Satisfaction with assistive Technology (QUEST).^52,53^
